# Anticipatory Violence and Health Among Black Adults in the United States

**DOI:** 10.1007/s40615-024-02257-w

**Published:** 2025-01-07

**Authors:** Daniel C. Semenza, Cortney VanHook, Nazsa S. Baker, Brielle Savage

**Affiliations:** 1 Department of Sociology, Anthropology, and Criminal Justice, Rutgers University – Camden, 405-7 Cooper Street, Camden, NJ 08102 USA; 2 Department of Urban-Global Public Health, Rutgers University, Piscataway, NJ USA; 3 New Jersey Gun Violence Research Center, Rutgers University, Piscataway, NJ USA; 4 School of Social Work, University of Illinois Urbana-Champaign, Champaign, IL USA; 5 School of Criminal Justice, Rutgers University – Newark, Newark, NJ USA; 6 San Francisco Wraparound Project, University of California - San Francisco, San Francisco, CA USA

**Keywords:** Anticipatory violence, Firearm violence, Police, Black Americans, Sleep, Physical health, Mental health

## Abstract

This study analyzes the relationship between anticipatory community and police violence and health outcomes including mental and physical well-being, sleep problems, and functional disability. Using data from a nationally representative survey of 3015 self-identified Black and African American adults in the USA collected in 2023, findings from a series of regression analyses reveal that anticipating community violence is linked to poorer self-rated health and increased sleep problems. Anticipatory police violence is associated with poorer physical health and sleep disturbances. These associations persist even after accounting for previous experiences of violence. The results underscore the potential health consequences of anticipating violence, suggesting that the fear of personal victimization can adversely influence health. Addressing anticipatory violence through trauma-informed public health policies and practices is critical for improving health outcomes and reducing disparities in violence-exposed communities. Future research should explore longitudinal impacts and extend analyses to additional racial groups and health outcomes.

The harmful effects of violence exposure extend beyond direct victimization and mounting evidence suggests the anticipation of violence may be associated with poorer health [1–3]. Drawing on Alang’s (2022) research regarding anticipatory stress and heightened vigilance related to police brutality [1], we define *anticipatory violence* here as the expectation of personal violent victimization at the hands of others. Anticipatory violence can include heightened fear of violent victimization in public or one’s local community, by intimate partners, and by the police. Anticipatory violence may stem from living in a neighborhood that experiences high rates of crime and greater perceived disorder [4]. It may also arise from previous direct or vicarious victimization (i.e., knowing someone personally who has been the victim of violence), generating psychological distress and activation of the stress response system harmful to health and well-being [5, 6]. Greater fear of being victimized, particularly in public or at the hands of the police, is likely to also influence important health behaviors such as walking or socializing outside that affect broader aspects of well-being [7].

Well-being, defined here broadly to include mental, physical, behavioral, and spiritual wellness as well as the absence of illness, is directly affected by stress responses within the body alongside environmental factors throughout the broader social world. The stress response system, also referred to as the hypothalamic–pituitary–adrenal (HPA) axis, is a central mechanism that manages stress and maintains homeostasis in the body. When the HPA axis is overburdened, it can lead to allostatic load, or a cumulative wear and tear on the body, due to repeated and prolonged activation of the stress response [8, 9]. Allostatic load derived from chronic activation of the stress response manifests in widespread harms to physical and mental well-being across all major systems of the body. Physical health outcomes associated with the stress response include high blood pressure [5], poor self-rated physical health [10], and deficits in working memory [11]. Anticipatory stressors are also associated with poorer self-rated mental health and psychiatric disorder diagnosis [1, 12]. Conflict within one’s social network and isolating behaviors are additional documented responses to anticipatory stress, which affect broader aspects of well-being [13, 14]. Regarding behavioral health, anticipatory stress can prevent people from leaving their homes, socializing with friends, exercising regularly, or engaging in healthy behaviors related to diet and sleep if they are concerned about the threat of violence in everyday life. This ultimately results in poorer quality of life and degraded functional health over time [15–17].

Despite growing research on actual experiences of violence exposure and well-being, there have been few studies to examine the specific relationship between anticipatory violence and diverse health outcomes, particularly among Black Americans, who are disproportionately exposed to community and police violence in the United States compared to other racial groups (US) [18, 19]. This is a notable omission given the extensive effects of racialization on well-being and the particular impacts of race-related stress on diverse aspects of health [20, 21]. Both the actual experience and the expectation of violence are shaped by intersecting processes of marginalization and systems of oppression such as structural racism that influence the risk of violence in everyday life, resulting in a much greater burden among Black Americans [22]. Thus, it is not one’s racial identity per se that enhances risk of violence, but rather structural conditions that lead to the racialization of certain groups and the resultant heightened exposure to violence that carries negative health consequences. Black individuals are much more likely than those belonging to other racial groups to be the victims of violence over the life course [23, 24]. An analysis of the Fragile Families and Child Wellbeing Study found that 56% of Black youth lived within 1300 meters of a gun homicide compared to 17% of White youth [24]. In the same study, roughly a quarter of Black youth surveyed had experienced three or more gun homicide incidents in their community compared to Hispanic (20%) and White (0.6%) youth. In a nationally representative sample of Black Americans, researchers recently found that 57% of Black adults have been exposed to at least one type of direct, secondary, or community-level gun violence in their life while about 12% have been exposed to three or more types [25]. Gun violence exposure among Black Americans is associated with an array of poorer health outcomes related to mental and physical well-being, suicidal ideation and attempt, sleep, and functional disability [17, 26, 27].

Black communities are also disproportionately harmed by the police. Black individuals are more likely to be stopped and arrested while driving [28], experience nonfatal police aggression [18], and are more likely to have militarized police units employed to their neighborhoods [29]. Black men and women are disproportionately killed by police use of force throughout the country [30]. Police brutality is a structural form of violence experienced disproportionately by marginalized communities and shaped by broader structures of racialization, thereby operating as a social determinant of health with implications for the development and perpetuation of disparities in well-being along racial lines [1, 31].

Both experienced and anticipatory violence in the community and by the police can differ in important ways, depending on demographic factors such as age, socioeconomic status, and geographical region [32, 33]. For example, gun violence is most commonly experienced by young Black men living in urban communities characterized by significant socioeconomic disadvantage, compared to other segments of Black populations as well as different racial groups [34, 35]. Both anticipated violence and actual exposure risk also differ across diverse Black groups such as Afro-Caribbeans, Africans, and African Americans [36, 37]. Thus, it is important to note that although Americans racialized as Black may experience a higher risk of violence in their lives, their experiences are not monolithic and likely to be diverse across populations. In this study, we draw on a national sample of self-identified Black and African American adults to examine how anticipatory violence related to one’s community and the police is associated with diverse health outcomes including mental and physical health, sleep, and everyday well-being related to functional disability.

## Data and Methods

### Data

To understand and measure experiences of gun violence exposure, health, and firearm behaviors, we conducted a nationally representative survey of Black adults in the US in April and May 2023 in collaboration with Ipsos Public Affairs KnowledgePanel, a probability-based web panel that represents all US adults. We conducted the survey specifically with self-identified Black adults given elevated rates of gun violence exposure alongside disproportionate experiences of police violence within this population in the US. After completing a demographic survey to confirm racial identification, members of the KnowledgePanel were invited by email to complete the study survey. Email reminders were sent to non-responders three days after first contact and every three days afterwards. For additional information about Ipsos KnowledgePanel’s sampling strategy and weighting methodology, please see https://www.ipsos.com/en-us/solutions/public-affairs/knowledgepanel.

The survey was completed by 3015 qualified KnowledgePanel respondents who self-identified as Black or African American. Respondents were asked, “Do you consider yourself to be (select all that apply): a) American Indian or Alaska Native, b) Asian, c) Black of African American, d) Caribbean Black, e) Indo Caribbean, f) Native Hawaiian or Other Pacific Islander, g) White, or h) Other (please specify).” All respondents who selected “Black or African American” were included. The International Review Board at Rutgers University approved the survey. The median time to complete the survey was 27 min and qualified KnowledgePanel respondents received a cash-equivalent incentive worth $5 for completing the survey [38].

To ensure the sample was representative of Black adults in the US, we employed post-stratification weights. These weights were developed by aligning the respondent pool with geodemographic benchmarks that merged a supplement of the Current Population Survey with the Census Bureau’s American Community Survey. The qualified respondents were weighted according to geodemographic distributions such as household income, gender, race, census region, metropolitan status, and education. The final weights were produced through an iterative raking procedure across all distributions, and they were scaled and trimmed to form the final sample.

### Measures

#### Health Outcomes

We examined five different health outcomes based on prior research demonstrating relationships between gun violence exposure and well-being among Black Americans [25–27]. These five outcomes were chosen to account for diverse aspects of mental, physical, and behavioral health that contribute to holistic well-being. S*elf-rated health* was measured using the question, “In general, how would you rate your health today?” Response categories included poor, fair, good, very good, and excellent, ranging from 1 through 5. This validated measure predicts a wide range of health outcomes and mortality [39].

We measured *physical health* by asking, “Thinking about your physical health, which includes physical illness and injury, for how many days during the past 30 days was your physical health not good?” Similarly, *mental health* was measured using the question, “Thinking about your mental health, which includes stress, depression, and problems with emotions, how many days during the past 30 days was your mental health not good?” We coded both measures into binary variables where respondents received a “1” if they indicated 14 or more days of poor mental or physical health and a “0” for fewer than 14 days. These two “health days” measures have been used frequently in the Behavioral Risk Factor Surveillance System (BRFSS) and the National Health and Nutrition Examination Survey (NHANES) run by the Centers for Disease Control [40].

We measured *sleep problems* by asking, “Please rate the current (i.e., last two weeks) severity of your sleep problems. Select the number that best represents your response.” This was asked for (a) difficulty falling asleep, (b) difficulty staying asleep, and (c) problems waking up too early. Responses ranged on a Likert scale from 0 (none) to 4 (very severe). Questions were derived from the validated Insomnia Severity Index [41]. We summed the three items to create an index of total sleep problems (alpha = 0.86).

We assessed *functional disability* using four items derived from the American Community Survey [42, 43]. Concentration difficulty was measured using the question, “Because of a physical, mental, or emotional condition, do you have serious difficulty concentrating, remembering, or making decisions?” Difficulty with walking/stairs was measured with the question, “Do you have serious difficult walking or climbing stairs?” We assessed difficulty dressing/bathing with the question, “Do you have difficulty dressing or bathing?” and difficulty doing everyday tasks like running errands by asking, “Because of a physical, mental, or emotional condition, do you have difficulty doing errands alone such as visiting a doctor’s office or shopping?” All responses were binary (0 = no; 1 = yes). The four items were added up to create a variety scale of total disability (range, 0–4).

#### Anticipatory Violence

We used a series of four questions to assess anticipatory violence as the main two exposure variables. Respondents were asked to indicate the likelihood of being: (a) shot by another person with a firearm, (b) physically attacked by another person without a firearm, (c) physically attacked by the police, and (d) shot by the police. For each, responses ranged from never (1) to very likely (4). We generated an average of the first two items to measure *anticipatory community violence* (alpha = 0.84) and an average of the two police-related items to measure *anticipatory police violence* (alpha = 0.95). These measures are distinctive from broad “fear of crime” measures often used in criminological research because they measure specific expectations of personal victimization by other citizens and the police rather than more generalized perceptions of criminal activity [44, 45].

#### Gun Violence Exposure

To account for prior experiences of gun violence exposure, we used four separate items derived from past studies on gun violence exposure and well-being [25–27]. *Shot with a firearm* was assessed with the question, “Have you ever been shot on purpose by another person with a firearm?” *Threatened with a firearm* was evaluated with the question, “Have you ever been threatened with a firearm by another person?” The item *family/friend shot* was measured using the question, “Do you personally know someone such as a friend or family member who has been shot on purpose by another person with a firearm?” Lastly, *witnessed/heard about a shooting* was assessed with the question, “Have you ever witnessed or heard about someone being shot intentionally by another person with a firearm in your neighborhood?” For all four items, respondents provided a “yes” or “no” response.

#### Covariates

We included a range of demographic covariates in all models including *sex* (male, female), *self-rated health* (poor, fair, good, very good, excellent), *age* (18–29, 30–44, 45–59, 60 +), *education* (no high school, high school degree, some college, bachelors, or more), *household income* (< $24,999, $25,000 to $74,999, $75,000 to $149,999, $150,000 +), *marital status* (married, widowed, divorced, separated, never married), *employment status* (working full time, working part time, not working), *current or former military status* (yes, no), health insurance (yes, no), *metropolitan area residence* (yes, no), *number of children living in the home*, and *US region of residence* (Northeast, Midwest, South, West). We accounted for *willingness to engage with the police* using an index comprised of three items. Respondents were asked to indicate how likely they would be to (a) call the police to report a crime occurring in your neighborhood, (b) help the police find someone suspected of committing a crime by providing them with information, and (c) report dangerous or suspicious activities in your neighborhood. Responses ranged from never (1) to very likely (4) (alpha = 0.89).

### Analytic Strategy

We first generated descriptive statistics for all measures, depicted in Table 1. To assess demographic differences in anticipatory violence, we conducted bivariate analyses for both types of anticipatory violence across four key demographic measures: age group, household income, education level, and region, depicted in the Appendix. The results demonstrate that levels of both community and police anticipatory violence were largely similar across demographic groups, although anticipatory police violence decreases as respondents age. We then ran five multivariate models dependent on the distribution of the health outcomes of interest. Ordinary least squares (OLS) regression was used to regress self-rated health on all independent and control measures given the variable’s normal distribution. We used logistic regression for the binary physical health and mental health days models, generating incidence odds ratios (ORs). Finally, we used negative binomial regression models for sleep problems and functional disability given the over-dispersed count nature of the two outcomes, generating incidence rate ratios (IRRs). We used listwise deletion to account for missing data across all variables included in our models (8% missing). All analyses were conducted using Stata 18.

**Table 1:** Weighted descriptive statistics (*N* = 2764)

	*N*	Percent
Poor physical health (14 + days)	337	12
Poor mental health (14 + days)	363	13
Gun violence exposure		
Threatened w/ gun	591	21
Shot w/ gun	69	3
Family/friend shot	1141	42
Witnessed/heard about shooting	1052	38
Female	1508	55
Age		
18–29	507	18
30–44	882	32
45–59	683	25
60 +	681	25
Education		
No HS	147	5
HS degree	995	36
Some college	860	31
Bachelors or more	750	27
Household income		
< $24,999	626	19
$25,000 to $74,999	1069	39
$75,000 to $149,999	779	28
$150,000 +	380	14
Marital status		
Married	995	36
Widowed	134	5
Divorced/separated	402	15
Never married	1221	44
Employment status		
Working full time	1476	54
Working part time	308	11
Not working	969	35
Health insurance	2505	91
Metropolitan status	2511	91
Region		
Northeast	453	16
Midwest	442	16
South	1572	57
West	287	10
	*M*	SD
Self-rated health	2.20	0.02
Sleep problems	3.02	0.08
Functional disability	0.42	0.02
Anticipatory violence (community)	0.64	0.02
Anticipatory violence (police)	0.82	0.03
Police engagement	9.70	0.08

## Results

Table 2 illustrates the multivariate results for self-rated, physical, and mental health. After accounting for prior gun violence exposure and all controls, anticipatory community violence was associated with a decrease in self-rated health (coef. = − 0.19, *p* < 0.001). However, no significant association with self-rated health was found for anticipatory police violence. Being threatened with a gun was the only experience of gun violence associated with poorer self-rated health (coef. = − 0.15, *p* < 0.05). Regarding physical health, anticipatory police violence was associated with a 24% increase in the odds of experiencing 14 + days of poor physical health in the past month (*p* < 0.05). We found no association between either measure of anticipatory violence or poor mental health. Witnessing or hearing shootings in one’s community was associated with substantially increased odds of both poor physical health (OR = 1.61, *p* < 0.01) and mental health (OR = 1.99, *p* < 0.001).

**Table 2:** Anticipatory violence and health (*N* = 2764)

	Self-rated health				Physical health				Mental health			
	Coef	SE	CI		OR	SE	CI		OR	SE	CI	
*Anticipatory violence*												
Community	− 0.19***	0.05	− 0.28	− 0.10	1.22	0.17	0.93	1.60	1.27	0.19	0.95	1.69
Police	− 0.04	0.04	− 0.11	0.03	1.24*	0.12	1.03	1.51	1.16	0.14	0.91	1.48
*Experienced gun violence*												
Threatened w/ gun	− 0.15*	0.07	− 0.28	− 0.01	1.13	0.23	0.76	1.67	1.20	0.27	0.77	1.87
Shot w/ gun	0.12	0.20	− 0.27	0.50	1.23	0.50	0.56	2.73	0.81	0.39	0.31	2.09
Family/friend shot	− 0.04	0.06	− 0.15	0.07	1.25	0.22	0.89	1.76	1.19	0.23	0.81	1.74
Witnessed/heard shooting	− 0.09	0.06	− 0.20	0.02	1.61**	0.28	1.15	2.28	1.99***	0.37	1.38	2.88

All models control for police engagement, sex, age, education, income, marital status, employment status, health insurance status, MSA status, and region

*OR* odds ratio

^***^p < .001; **p < .01; *p < .05

Table 3 shows the multivariate results for sleep problems and functional disability. Both anticipatory community (IRR = 1.14, *p* < 0.01) and police (IRR = 1.08, *p* < 0.05) violence were associated with a greater incidence rate of sleep problems. Additionally, being threatened with a gun (IRR = 1.17, *p* < 0.05), knowing a family member or friend that has been shot (IRR = 1.14, *p* < 0.05), and witnessing or hearing shootings in one’s community (IRR = 1.23, *p* < 0.001) were all associated with greater sleep problems. On the other hand, we found no association between either measure of anticipatory violence or functional disability. Being threatened with a gun (IRR = 1.36, *p* < 0.01) and witnessing or hearing about shootings in one’s community (IRR = 1.27, *p* < 0.05) were associated with an increased incidence rate of functional disability.

**Table 3:** Anticipatory violence, sleep problems, and functional disability (*N* = 2764)

	Sleep problems				Functional disability			
	IRR	SE	CI		IRR	SE	CI	
*Anticipatory violence*								
Community	1.14**	0.05	1.05	1.24	1.17	0.10	0.99	1.39
Police	1.08*	0.04	1.01	1.15	1.13	0.07	0.99	1.28
*Experienced gun violence*								
Threatened w/ gun	1.17*	0.07	1.04	1.33	1.36**	0.16	1.08	1.71
Shot w/ gun	1.07	0.15	0.81	1.41	1.26	0.35	0.73	2.19
Family/friend shot	1.14*	0.07	1.02	1.28	1.19	0.13	0.96	1.49
Witnessed/heard shooting	1.23***	0.07	1.09	1.37	1.27*	0.14	1.02	1.58

All models control for police engagement, sex, age, education, income, marital status, employment status, health insurance status, MSA status, and region

*IRR* incidence rate ratio

^***^p < .001; **p < .01; *p < .05

## Discussion

We examined associations between anticipatory community and police violence and a range of health outcomes among self-identified Black and African American adults in the US. Our results produced two key findings. First, anticipating community violence was associated with poorer self-rated health and more sleep problems. Second, anticipating police violence was linked to poorer physical health and more sleep problems. Overall, we found that anticipatory violence was associated with poorer health even when accounting for actual prior experiences of violence, suggesting that the expectation of violence may uniquely and adversely influence the health of Black Americans.

Anticipatory violence is heightened in Black communities, which are disproportionately affected by both community violence and police brutality. Although research has often focused on health-related consequences tied to direct victimization [46], offending [47, 48], or witnessed violence [49], many people also express concerns about anticipatory violence [50]. Communities disproportionately exposed to police presence also experience brutality vicariously or indirectly through others in their social networks or the media [12]. Those racialized as Black in the US are more subject to a range of structurally discriminatory practices that converge to influence health and well-being across a spectrum of outcomes. The association between anticipatory community violence and poorer self-rated health and sleep underscores the deep impact that fear of violence in one’s own community can have on Black adults. Black adults already experience significantly poorer health across a range of domains compared to their White counterparts, and it is possible that anticipatory violence exacerbates existing health-related inequalities. For instance, Black Americans experience disproportionately higher rates of hypertension, diabetes, cardiovascular disease, and stroke when compared to other racial and ethnic groups in the US [51], many of which are directly associated with anticipatory stress responses, although these differences vary across culturally distinct groups racialized as Black (e.g., Afro-Caribbean, Black Europeans).

Additionally, the results revealed a significant association between anticipating police violence and both poorer physical health and sleep problems. These findings support existing literature on the relationship between violence exposure and sleep disturbances [52–54]. Our findings also cohere with a growing body of research showing that both direct involvement in police stops and witnessing intrusive stops by the police are harmful to diverse aspects of well-being, including sleep, especially among youth of color [55–57]. Research shows that Black Americans are at greater risk of police violence and excessive use of force, and our results suggest that merely anticipating such violence may be detrimental to the overall health of Black Americans [58].

Notably, the results did not reveal evidence of an association between anticipatory violence and poor mental health, potentially due to the high threshold nature of our measure (i.e., 14 + days of poor mental health in a month). Still these results are somewhat surprising given that it is well documented that police brutality is associated with negative health outcomes and has harmful effects among Black individuals [59, 60]. Yet there may be explanations for the lack of findings beyond the way that mental health was measured in this study. Survey respondents may have underreported consistently poor feelings of mental health due to greater stigma around mental illness documented among Black individuals and other racially minoritized groups [61, 62]. On the other hand, the well-documented John Henryism effect shown to be harmful to the physical health of Black Americans may result in more active, robust coping efforts that protect Black individuals from the psychological harms of anticipatory violence if they have become inured to the effects of these experiences [63, 64]. This may explain the null findings when exploring the specific effects of anticipatory violence on mental well-being. Despite the pervasive nature of violence exposure in urban communities in the US, research on broader mental health consequences of community violence among adults and other older age groups remains sparse, which has substantial public health significance and suggests the need for continued research in this area [65].

This study has certain limitations that provide opportunities for future research. First, the cross-sectional nature of the data precludes causal claims about the relationship between anticipatory violence and well-being. Longitudinal research can illustrate how anticipating violence at a given time point shapes health outcomes over time. Second, our results are only generalizable to those who identify as Black or African American adults in the US. Researchers should aim to replicate these analyses using a national sample that spans racial groups to assess whether these results differ among non-Black populations, as well as assess differences between culturally distinct populations among people racialized as Black including naturalized Africans, Afro-Caribbeans, Black Europeans, and Canadian Black groups. More research is similarly needed on the relationship between gun violence exposure and diverse health outcomes among youth of all races. Finally, our analyses were limited to available health measures as the dependent variables. Further research is necessary to examine whether anticipatory violence by others in the community and the police is associated with additional aspects of health such as substance use, exercise, diet, and chronic illness.

Despite these limitations, our findings underscore the need for greater efforts to directly address anticipatory community and police violence as a means of improving broader health outcomes, particularly among communities of color most impacted by violence [66]. Conceptualizing anticipatory violence as a driver of health disparities supports continued efforts for trauma-informed research, policy, and practice that focus on Black Americans. Significantly reducing violence both within communities and by the police is essential for improving many aspects of public health and safety [67]. Our finding that anticipatory violence is linked to poorer health above and beyond actual experiences of violence suggests the health consequences of violence exposure reverberate far beyond direct victimization [68]. Ultimately, intersectional public health approaches that combine prevention, intervention, and culturally congruent treatment strategies are necessary to mitigate the harms of anticipatory effects of both police and community violence for Black adults.

## Figures and Tables

**Fig. 1:**
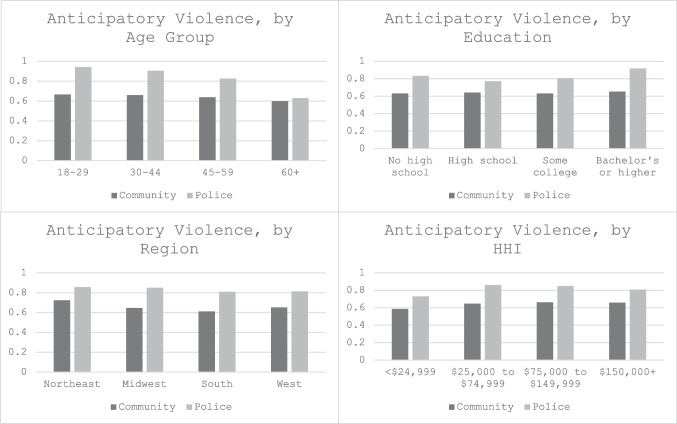
Anticipatory violence by demographic groups

## Data Availability

Data are available from the authors upon request.

## Appendix

Figure 1
